# Supplementary material for: Factors influencing IMF assistance in the Sub-Saharan African region
Source: PLoS One. 2024 Jul 16;19(7):e0307071. doi: 10.1371/journal.pone.0307071 (PMC11251602; doi:10.1371/journal.pone.0307071)
Supplement: S2 Appendix — (DOCX) [file pone.0307071.s002.docx]

**S2 Appendix: Summary descriptive statistics of each country.**

| **No** | **Country** | **Variables** | | | | | | |
| --- | --- | --- | --- | --- | --- | --- | --- | --- |
|  |  |  | **CAB** | **GGNLB** | **GGGD** | **INFLATION** | **CORRUPTION** | **GDPG** |
| **1** | Angola | Obs. | 23 | 23 | 23 | 23 | 23 | 23 |
|  |  | Mean | 4.328174 | 0.448522 | 3.989906 | 38.98057 | -1.26853 | 4.716526 |
|  |  | SD | 8.732586 | 4.793127 | .05858896 | 58.25169 | 0.184908 | 5.706967 |
|  |  | Min. | -13.092 | -7.897 | 2.930073 | 7.498 | -1.46788 | -5.63822 |
|  |  | Max. | 20.408 | 9.423 | 4.933733 | 268.35 | -0.65535 | 15.03 |
| **2** | Benin | Obs. | 23 | 23 | 23 | 23 | 23 | 23 |
|  |  | Mean | -4.57326 | -2.34022 | 3.297252 | 2.248957 | -0.55698 | 4.67888 |
|  |  | SD | 1.485557 | 1.976426 | .4533468 | 2.934675 | 0.212886 | 1.759215 |
|  |  | Min. | -6.69 | -5.709 | 2.124176 | -2.761 | -0.87133 | 1.713165 |
|  |  | Max. | -1.746 | 0.222 | 3.958639 | 8.936 | -0.03325 | 7.191434 |
| **3** | Botswana | Obs. | 23 | 23 | 23 | 23 | 23 | 23 |
|  |  | Mean | 3.970739 | -0.89126 | 2.555029 | 6.803391 | 0.878768 | 3.463461 |
|  |  | SD | 7.868186 | 6.784068 | .4559028 | 3.327196 | 0.162527 | 6.04218 |
|  |  | Min. | -8.654 | -13.78 | 1.780193 | 2.153 | 0.624538 | -14.1442 |
|  |  | Max. | 19.665 | 13.311 | 3.040992 | 13.678 | 1.24492 | 11.87036 |
| **4** | Burkina Faso | Obs. | 23 | 23 | 23 | 23 | 23 | 23 |
|  |  | Mean | -5.87309 | -3.36078 | 3.443147 | 2.509696 | -0.22121 | 5.358976 |
|  |  | SD | 3.8752 | 4.33438 | .2991055 | 3.462065 | 0.171931 | 2.001817 |
|  |  | Min. | -10.766 | -10.385 | 3.000172 | -2.556 | -0.5253 | 1.479301 |
|  |  | Max. | 4.144 | 14.346 | 3.99469 | 11.578 | 0.124697 | 8.661873 |
| **5** | Burundi | Obs. | 23 | 23 | 23 | 23 | 23 | 23 |
|  |  | Mean | -12.841 | -6.72991 | 4.273925 | 9.270043 | -1.20736 | 2.398373 |
|  |  | SD | 6.268681 | 3.711491 | .5893811 | 7.202852 | 0.253177 | 2.494156 |
|  |  | Min. | -32.41 | -15.46 | 3.246102 | -5.877 | -1.5964 | -3.9 |
|  |  | Max. | -3.508 | -1.899 | 5.151776 | 26.642 | -0.7599 | 5.413807 |
| **6** | Cabo Verde | Obs. | 23 | 23 | 23 | 23 | 23 | 23 |
|  |  | Mean | -9.163 | -6.11187 | 4.550551 | 2.024913 | 0.819956 | 4.619578 |
|  |  | SD | 4.485241 | 3.800152 | .2648617 | 2.763205 | 0.192023 | 7.154989 |
|  |  | Min. | -16.287 | -17.84 | 4.143357 | -2.281 | 0.338964 | -19.3028 |
|  |  | Max. | 0.208 | -0.941 | 4.977602 | 7.577 | 1.127157 | 17.71418 |
| **7** | Cameroon | Obs. | 23 | 23 | 23 | 23 | 23 | 23 |
|  |  | Mean | -2.74822 | 0.015087 | 3.384961 | 2.43187 | -1.13704 | 3.964043 |
|  |  | SD | 1.415242 | 6.678018 | .6049006 | 1.526745 | 0.083614 | 1.384668 |
|  |  | Min. | -4.273 | -5.881 | 2.411709 | -0.055 | -1.32529 | 0.259933 |
|  |  | Max. | 1.195 | 28.214 | 4.329166 | 6 | -0.98134 | 7.048863 |
| **8** | CAR | Obs. | 23 | 23 | 23 | 23 | 23 | 23 |
|  |  | Mean | -6.44287 | -1.45213 | 3.943337 | 4.235391 | -1.16112 | 1.203324 |
|  |  |  |  |  |  |  |  |  |
|  |  | SD | 3.655902 | 3.064929 | .5119953 | 5.054061 | 0.13262 | 8.714283 |
|  |  | Min. | -13.324 | -6.017 | 2.979908 | -4.466 | -1.47727 | -36.392 |
|  |  | Max. | -1.457 | 8.584 | 4.635845 | 14.525 | -0.93761 | 8.58726 |
| **9** | Chad | Obs. | 23 | 23 | 23 | 23 | 23 | 23 |
|  |  | Mean | -11.4441 | -1.37257 | 3.663475 | 2.944304 | -1.40346 | 5.645199 |
|  |  | SD | 19.34005 | 3.653592 | .3375133 | 6.197552 | 0.065951 | 8.489678 |
|  |  | Min. | -84.105 | -9.203 | 3.00067 | -11.864 | -1.53419 | -6.25553 |
|  |  | Max. | 8.15 | 5.145 | 4.219287 | 13.046 | -1.27031 | 33.62937 |
| **10** | Comoros | Obs. | 23 | 23 | 23 | 23 | 23 | 23 |
|  |  | Mean | -2.39522 | -0.46583 | 3.313812 | 3.615217 | -0.81699 | 2.985776 |
|  |  | SD | 2.010491 | 3.222824 | .4780629 | 4.478829 | 0.171076 | 2.054343 |
|  |  | Min. | -6.059 | -5.52 | 2.329908 | -4.773 | -1.28728 | -0.19556 |
|  |  | Max. | 1.848 | 10.489 | 4.107886 | 17.084 | -0.58659 | 10.84788 |
| **11** | Cote d'Ivoire | Obs. | 23 | 23 | 23 | 23 | 23 | 23 |
|  |  | Mean | -0.10165 | -2.03617 | 3.79863 | 2.386261 | -0.82444 | 3.672043 |
|  |  | SD | 3.09058 | 1.755034 | .3332796 | 1.816447 | 0.324143 | 4.469463 |
|  |  | Min. | -6.535 | -6.726 | 3.203722 | -0.123 | -1.29001 | -5.37045 |
|  |  | Max. | 7.52 | 0.699 | 4.303889 | 5.579 | -0.37189 | 10.76021 |
| **12** | Eswatini | Obs. | 23 | 23 | 23 | 23 | 23 | 23 |
|  |  | Mean | 1.324478 | -3.031 | 2.975545 | 6.366913 | -0.16802 | 3.059191 |
|  |  | SD | 6.610612 | 4.292756 | .4413953 | 2.83116 | 0.149837 | 2.114506 |
|  |  | Min. | -11.572 | -9.538 | 2.332435 | 2.003 | -0.65057 | -1.55964 |
|  |  | Max. | 12.967 | 8.418 | 3.814476 | 12.904 | 0.036358 | 7.883307 |
| **13** | Ethiopia | Obs. | 23 | 23 | 23 | 23 | 23 | 23 |
|  |  | Mean | -5.74696 | 4.031242 | 4.031242 | 14.98926 | -0.59809 | 8.513311 |
|  |  | SD | 3.105076 | 0.379998 | .3799984 | 11.7191 | 0.160264 | 3.620465 |
|  |  | Min. | -11.71 | 3.399863 | 3.399863 | -4.3 | -0.90676 | -2.16136 |
|  |  | Max. | -0.674 | 4.676216 | 4.676216 | 39.245 | -0.35321 | 13.5726 |
| **14** | Gabon | Obs. | 23 | 23 | 23 | 23 | 23 | 23 |
|  |  | Mean | 7.107261 | 3.54487 | 3.823472 | 1.864957 | -0.81606 | 2.125868 |
|  |  | SD | 10.88284 | 4.672086 | .4753148 | 1.999706 | 0.182396 | 3.02165 |
|  |  | Min. | -11.063 | -4.71 | 2.998379 | -1.151 | -1.16633 | -3.30843 |
|  |  | Max. | 24.005 | 11.132 | 4.395313 | 6.253 | -0.52038 | 7.091753 |
| **15** | Ghana | Obs. | 23 | 23 | 23 | 23 | 23 | 23 |
|  |  | Mean | -4.6197 | -6.25813 | 3.822628 | 16.15387 | -0.1373 | 5.6735 |
|  |  | SD | 2.508936 | 3.599065 | .4315562 | 10.92682 | 0.112324 | 2.858548 |
|  |  | Min. | -8.953 | -17.443 | 2.918257 | 6.868 | -0.36875 | 0.513942 |
|  |  | Max. | 0.082 | -2.01 | 4.486015 | 54.1 | 0.038148 | 14.04712 |
| **16** | Guinea | Obs. | 23 | 23 | 23 | 23 | 23 | 23 |
|  |  | Mean | -8.95117 | -1.49796 | 4.004403 | 13.2923 | -1.03435 | 4.493681 |
|  |  | SD | 7.972401 | 5.904607 | .3965457 | 8.670917 | 0.119479 | 2.648531 |
|  |  | Min. | -30.656 | -9.661 | 3.302665 | 1.13 | -1.29245 | -1.12264 |
|  |  | Max. | 0.326 | 23.232 | 4.5841 | 39.132 | -0.76705 | 10.82063 |
| **17** | Guinea-Bissau | Obs. | 23 | 23 | 23 | 23 | 23 | 23 |
|  |  |  |  |  |  |  |  |  |
|  |  | Mean | -2.92144 | -3.62 | 4.58746 | 2.980522 | -1.29141 | 3.068477 |
|  |  | SD | 3.11756 | 2.764695 | .5872435 | 4.722936 | 0.191451 | 2.608739 |
|  |  | Min. | -8.544 | -9.638 | 3.836027 | -6.357 | -1.58114 | -2.4 |
|  |  | Max. | 2.725 | 2.679 | 5.380538 | 16.684 | -0.91532 | 8.08478 |
| **18** | Kenya | Obs. | 23 | 23 | 23 | 23 | 23 | 23 |
|  |  | Mean | 0.816174 | -4.33504 | 3.794948 | 7.666043 | -0.95485 | 4.271488 |
|  |  | SD | 9.775678 | 2.763209 | .2247019 | 4.334896 | 0.104041 | 2.263476 |
|  |  | Min. | -14.525 | -9.34 | 3.532079 | 1.602 | -1.14436 | -0.27277 |
|  |  | Max. | 20.36 | 0.619 | 4.218566 | 18.934 | -0.71327 | 8.058474 |
| **19** | Lesotho | Obs. | 23 | 23 | 23 | 23 | 23 | 23 |
|  |  | Mean | 0.816174 | -0.21365 | 3.88784 | 6.133522 | -0.00268 | 2.094013 |
|  |  | SD | 9.775678 | 5.597805 | .292469 | 2.363809 | 0.145921 | 2.981769 |
|  |  | Min. | -14.525 | -9.879 | 3.562522 | 2.012 | -0.31528 | -5.6194 |
|  |  | Max. | 20.36 | 11.553 | 4.688389 | 10.906 | 0.279654 | 6.334822 |
| **20** | Liberia | Obs. | 23 | 23 | 23 | 23 | 23 | 23 |
|  |  | Mean | -14.127 | -2.09239 | 4.558394 | 10.81578 | -0.8251 | 2.340424 |
|  |  | SD | 10.10743 | 2.899223 | 1.329333 | 5.627048 | 0.220207 | 7.95936 |
|  |  | Min. | -34.307 | -7.134 | 3.02062 | 3.204 | -1.32961 | -30.1451 |
|  |  | Max. | 2.218 | 4.231 | 6.397125 | 28.525 | -0.47649 | 9.535275 |
| **21** | Madagascar | Obs. | 23 | 23 | 23 | 23 | 23 | 23 |
|  |  | Mean | -6.28535 | -2.81844 | 3.844953 | 8.536565 | -0.57165 | 2.76438 |
|  |  | SD | 4.901628 | 1.44102 | .3954629 | 5.133286 | 0.387235 | 4.767506 |
|  |  | Min. | -18.172 | -6.764 | 3.339676 | -0.79 | -1.19242 | -12.408 |
|  |  | Max. | 0.716 | -0.76 | 4.502162 | 27.327 | 0.040672 | 9.784892 |
| **22** | Malawi | Obs. | 23 | 23 | 23 | 23 | 23 | 23 |
|  |  | Mean | -8.74778 | -3.52961 | 3.691371 | 15.67465 | -0.60214 | 3.970473 |
|  |  | SD | 3.874031 | 2.72921 | .5163792 | 8.796117 | 0.193543 | 3.0627 |
|  |  | Min. | -15.529 | -10.359 | 2.885359 | 6.27 | -1.02162 | -4.97496 |
|  |  | Max. | -1.435 | 0.625 | 4.620108 | 35.445 | -0.22871 | 9.599999 |
| **23** | Mali | Obs. | 23 | 23 | 23 | 23 | 23 | 23 |
|  |  | Mean | -6.42644 | -1.64317 | 3.554869 | 2.396348 | -1.64317 | -0.683 |
|  |  | SD | 2.965503 | 6.519421 | .4298396 | 3.331558 | 6.519421 | 0.118633 |
|  |  | Min. | -13.542 | -5.373 | 2.894032 | -4.987 | -5.373 | -0.87851 |
|  |  | Max. | -2.046 | 27.783 | 4.505593 | 8.847 | 27.783 | -0.42621 |
| **24** | Mauritius | Obs. | 23 | 23 | 23 | 23 | 23 | 23 |
|  |  | Mean | -5.34813 | -3.91413 | 4.12854 | 4.682783 | 0.303189 | 3.45708 |
|  |  | SD | 5.114182 | 1.972682 | .1751122 | 3.118402 | 0.111826 | 4.276226 |
|  |  | Min. | -13.49 | -10.442 | 3.88689 | 0.239 | 0.136082 | -14.5974 |
|  |  | Max. | 5.652 | -1.615 | 4.549932 | 12.193 | 0.48235 | 8.690735 |
| **25** | Mozambique | Obs. | 23 | 23 | 23 | 23 | 23 | 23 |
|  |  | Mean | -21.0575 | -3.77504 | 4.192181 | 9.065783 | -0.6359 | 5.909735 |
|  |  | SD | 11.76981 | 2.059583 | .4688544 | 5.775371 | 0.150102 | 3.016644 |
|  |  | Min. | -41.527 | -9.895 | 3.473735 | 1.925 | -0.88365 | -1.19855 |
|  |  | Max. | -6.281 | 0.112 | 4.837773 | 21.935 | -0.42872 | 12.08687 |
| **26** | Namibia | Obs. | 23 | 23 | 23 | 23 | 23 | 23 |
|  |  | Mean | -2.36922 | -3.59896 | 3.444208 | 6.106739 | 0.301488 | 3.27239 |
|  |  | SD | 7.826598 | 4.134884 | .4687996 | 3.131396 | 0.147141 | 3.85964 |
|  |  | Min. | -16.502 | -9.303 | 2.763232 | 2.361 | 0.114023 | -8.1014 |
|  |  | Max. | 14.043 | 6.007 | 4.276374 | 14.254 | 0.773133 | 12.26955 |
| **27** | Niger | Obs. | 23 | 23 | 23 | 23 | 23 | 23 |
|  |  | Mean | -10.6544 | -1.65965 | 3.481702 | 2.005087 | -0.7104 | 5.120817 |
|  |  | SD | 4.108648 | 7.413546 | .5808579 | 3.501214 | 0.143363 | 3.138967 |
|  |  | Min. | -18.097 | -6.877 | 2.651127 | -4.299 | -1.02049 | -1.20848 |
|  |  | Max. | -3.782 | 31.045 | 4.407987 | 13.594 | -0.5036 | 11.5 |
| **28** | Nigeria | Obs. | 23 | 23 | 23 | 23 | 23 | 23 |
|  |  | Mean | 4.476261 | -1.32661 | 3.092174 | 13.14352 | -1.15827 | 5.145792 |
|  |  | SD | 6.364929 | 4.369789 | .6210752 | 4.291024 | 0.148982 | 3.681069 |
|  |  | Min. | -3.723 | -6.029 | 1.984581 | 6.607 | -1.50207 | -1.79425 |
|  |  | Max. | 21.533 | 8.759 | 4.053523 | 23.811 | -0.90095 | 15.32916 |
| **29** | Rwanda | Obs. | 23 | 23 | 23 | 23 | 23 | 23 |
|  |  | Mean | -7.80096 | -2.1573 | 3.685568 | 6.43313 | 0.183857 | 7.417185 |
|  |  | SD | 3.785474 | 2.769414 | .5909267 | 5.829646 | 0.546413 | 3.347157 |
|  |  | Min. | -15.313 | -9.533 | 2.906518 | -0.219 | -0.7336 | -3.37399 |
|  |  | Max. | -1.846 | 2.274 | 4.523157 | 22.323 | 0.992052 | 13.19207 |
| **30** | SaSao Tome | Obs. | 23 | 23 | 23 | 23 | 23 | 23 |
|  |  | Mean | -13.7212 | 5.821739 | 4.791712 | 12.60691 | -0.07301 | 4.556413 |
|  |  | SD | 22.44101 | 30.59117 | .697371 | 6.852258 | 0.246875 | 2.118849 |
|  |  | Min. | -33.94 | -19.257 | 4.062149 | 4.183 | -0.54106 | 0.927137 |
|  |  | Max. | 84.849 | 125.135 | 6.016503 | 27.559 | 0.346064 | 8.866109 |
| **31** | Senegal | Obs. | 23 | 23 | 23 | 23 | 23 | 23 |
|  |  | Mean | -7.4283 | -3.14139 | 3.736108 | 2.207261 | -0.16171 | 4.120909 |
|  |  | SD | 3.172829 | 2.066996 | .4207372 | 3.331253 | 0.238631 | 1.913966 |
|  |  | Min. | -16.002 | -6.405 | 2.861458 | -4.545 | -0.64139 | 0.068697 |
|  |  | Max. | -3.522 | 0.782 | 4.317088 | 12.8 | 0.247914 | 7.407486 |
| **32** | Seychelles | Obs. | 23 | 23 | 23 | 23 | 23 | 23 |
|  |  | Mean | -14.0107 | -1.91691 | 4.601903 | 5.814869 | 0.769798 | 2.834934 |
|  |  | SD | 6.889271 | 6.789402 | .4604274 | 13.09716 | 0.372564 | 5.015647 |
|  |  | Min. | -24.597 | -16.262 | 3.992773 | -2.538 | 0.281312 | -8.64921 |
|  |  | Max. | -1.327 | 7.882 | 5.297252 | 63.252 | 1.633352 | 9.509784 |
| **33** | Sierra Leone | Obs. | 23 | 23 | 23 | 23 | 23 | 23 |
|  |  | Mean | -14.2267 | -3.66344 | 4.282423 | 10.43043 | -0.79214 | 5.457078 |
|  |  | SD | 12.92932 | 5.681646 | .5444577 | 8.072976 | 0.217887 | 8.788124 |
|  |  | Min. | -65.031 | -10.905 | 3.42064 | -3.08 | -1.08549 | -20.4911 |
|  |  | Max. | -5.043 | 20.09 | 5.196595 | 37.09 | -0.37105 | 26.52414 |
| **34** | South Africa | Obs. | 23 | 23 | 23 | 23 | 23 | 23 |
|  |  | Mean | -2.14226 | -3.02322 | 3.666354 | 5.631304 | 0.108944 | 2.35153 |
|  |  | SD | 2.375814 | 2.445143 | .3252474 | 2.450593 | 0.222912 | 2.603214 |
|  |  | Min. | -5.326 | -9.631 | 3.179927 | 0.26 | -0.18417 | -6.34247 |
|  |  | Max. | 3.677 | 1.219 | 4.262905 | 12.245 | 0.55027 | 5.603806 |
| **35** | Sudan | Obs. | 23 | 23 | 23 | 23 | 23 | 23 |
|  |  | Mean | -7.96974 | -3.11291 | 4.682626 | 48.78213 | -1.33281 | 1.491493 |
|  |  | SD | 4.305122 | 3.084757 | .424828 | 80.7295 | 0.163853 | 5.4309 |
|  |  | Min. | -17.45 | -10.822 | 3.983264 | 1.81 | -1.54606 | -17.0047 |
|  |  | Max. | -2.625 | 0.768 | 5.616902 | 318.208 | -1.01889 | 6.531412 |
| **36** | Tanzania | Obs. | 23 | 23 | 23 | 23 | 23 | 23 |
|  |  | Mean | -5.5393 | -2.56435 | 3.587912 | 6.521783 | -0.55311 | 5.980282 |
|  |  | SD | 3.26597 | 1.211606 | .2352241 | 4.036028 | 0.175343 | 1.327271 |
|  |  | Min. | -11.565 | -4.744 | 3.079292 | 3.154 | -0.84093 | 1.991965 |
|  |  | Max. | -1.131 | -0.414 | 3.927306 | 19.754 | -0.22858 | 7.672155 |
| **37** | Togo | Obs. | 23 | 23 | 23 | 23 | 23 | 23 |
|  |  | Mean | -4.96813 | -2.92465 | 4.004199 | 2.357217 | -0.86643 | 3.76434 |
|  |  | SD | 2.509842 | 2.95748 | .2155017 | 2.782167 | 0.139587 | 3.11774 |
|  |  | Min. | -9.002 | -7.369 | 3.535757 | -1.675 | -1.04961 | -4.6663 |
|  |  | Max. | -0.274 | 1.613 | 4.301386 | 8.698 | -0.668 | 6.720171 |
| **38** | Uganda | Obs. | 23 | 23 | 23 | 23 | 23 | 23 |
|  |  | Mean | -4.74317 | -2.68735 | 3.448539 | 6.152783 | -0.97198 | 5.90955 |
|  |  | SD | 2.681932 | 2.141517 | .450764 | 5.912976 | 0.106668 | 2.180193 |
|  |  | Min. | -9.468 | -7.494 | 2.694154 | -4.424 | -1.14086 | 2.951306 |
|  |  | Max. | 0.395 | 0.343 | 4.009857 | 27.009 | -0.78762 | 10.78474 |
| **39** | Zambia | Obs. | 23 | 23 | 23 | 23 | 23 | 23 |
|  |  | Mean | -0.85757 | -4.13122 | 4.041748 | 13.33809 | -0.5322 | 5.375435 |
|  |  | SD | 7.099781 | 5.751759 | .8714738 | 6.747252 | 0.153708 | 2.813328 |
|  |  | Min. | -17.426 | -13.783 | 2.939056 | 6.085 | -0.81826 | -2.78506 |
|  |  | Max. | 10.613 | 16.913 | 5.564383 | 30.14 | -0.29144 | 10.29822 |
