# Supplementary material for: Factors influencing IMF assistance in the Sub-Saharan African region
Source: PLoS One. 2024 Jul 16;19(7):e0307071. doi: 10.1371/journal.pone.0307071 (PMC11251602; doi:10.1371/journal.pone.0307071)
Supplement: S3 Appendix — (DOCX) [file pone.0307071.s003.docx]

| **Variable** | **Specification** | | | | | | |  |  |  |  |  |
| --- | --- | --- | --- | --- | --- | --- | --- | --- | --- | --- | --- | --- |
|  | 1 | 2 | 3 | 4 | 5 | 6 | 7 | | 8 | 9 | 10 | 11 |
| Constant | -1.497 | -1.370 | -1.503 | -1.423 | -2.111 | -2.075 | -2.193 | | -2.179 | -2.170 | -2.170 | -2.299 |
| Corruption  GDPG  ED  China  Ln GGGD  GGNLB  EA  UNSC  Inflation  CAB  LD | -0.391^a^ | -0.424^a^  -0.046^a^ | -0.420^a^  -0.046^a^  0.379^a^ | -0.469^a^  -0.044^a^  0.379^a^  -0.236^a^ | -0.471^a^  -0.040^a^  0.382^a^  -0.210^a^  0.175^a^ | -0.475^a^  -0.037^a^  0.379^a^  -0.209^a^  0.150^b^  -0.017^b^ | -0.428^a^  -0.037^a^  0.505^a^  -0.213^a^  0.155^a^  -0.017^b^  0.208^c^ | | -0.424^a^  -0.037^a^  0.505^a^  -0.217^a^  0.155^a^  -0.018^b^  0.206^c^  -0.208 | -0.412^a^  -0.037^a^  0.511^a^  -0.214^a^  0.146^b^  -0.018^b^  0.217^c^  -0.205  0.003 | -0.410^a^  -0.037^a^  0.499^a^  -0.214^a^  0.146^b^  -0.016^b^  0.211^c^  -0.200  0.003  -0.004 | -0.441^a^  -0.037^a^  0.598^a^  -0.212^a^  0.150^b^  -0.016^b^  0.290^c^  -0.202  0.003  -0.004  0.197 |
| lnsig^2^u | -1.35 | -1.25 | -1.42 | -1.28 | -1.33 | -1.32 | -1.37 | | -1.37 | -1.35 | -1.38 | -1.36 |
| Wald chi^2^ | 44.32 | 92.84 | 111.37 | 116.88 | 125.04 | 126.45 | 129.08 | | 130.64 | 81.87 | 132.64 | 132.61 |
| Log-likelihood | -1145.6 | -1118.8 | -1108.9 | -1103.8 | -1099.2 | -1096.6 | -1095.2 | | -1094.0 | -1093.3 | -1092.8 | -1092.3 |
| LR test chibar^2^ | 95.09 | 102.88 | 81.00 | 89.90 | 85.94 | 86.54 | 80.29 | | 80.78 | 81.87 | 78.19 | 79.05 |
| No of Obs | 3,680 | 3,680 | 3,680 | 3,680 | 3,680 | 3,680 | 3,680 | | 3,680 | 3,680 | 3,680 | 3,680 |

**S3 Appendix: Panel probit estimated results and marginal effect in percentage.**

Note: The coefficients presented above represent marginal effect with the significant levels as follows, a significant at 1%, b at 5%, and c significant 10% significance level.
