# Supplementary figures and images for: Factors influencing IMF assistance in the Sub-Saharan African region
Source: PLoS One. 2024 Jul 16;19(7):e0307071. doi: 10.1371/journal.pone.0307071 (PMC11251602; doi:10.1371/journal.pone.0307071)

**S4 : Appendix: Line graph analysis of macroeconomic variables across the SSA region**


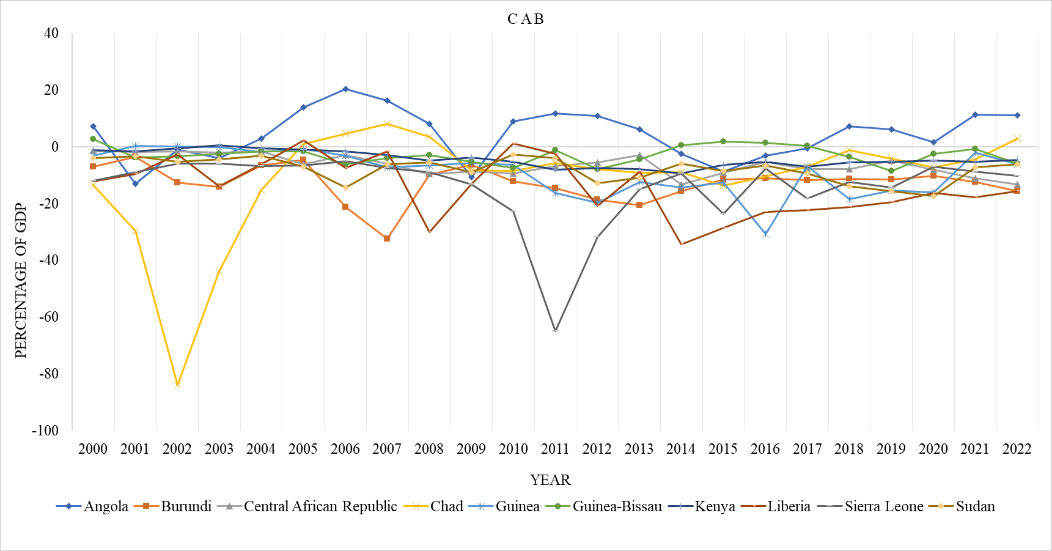

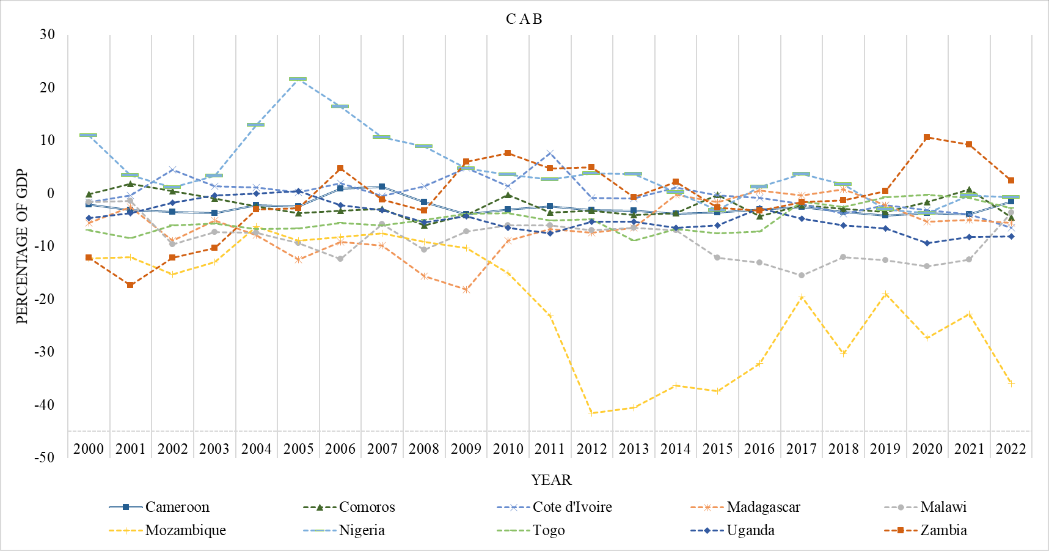

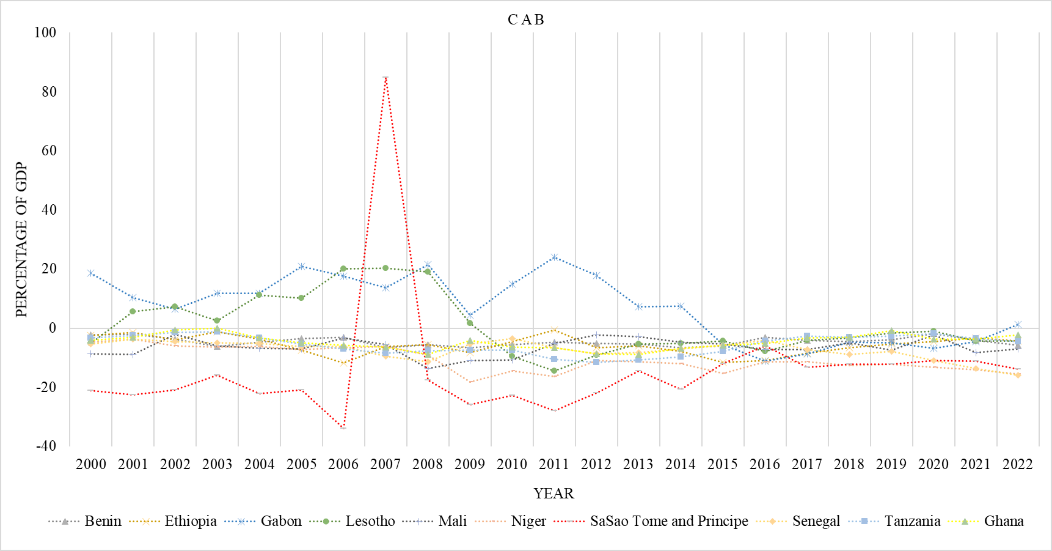

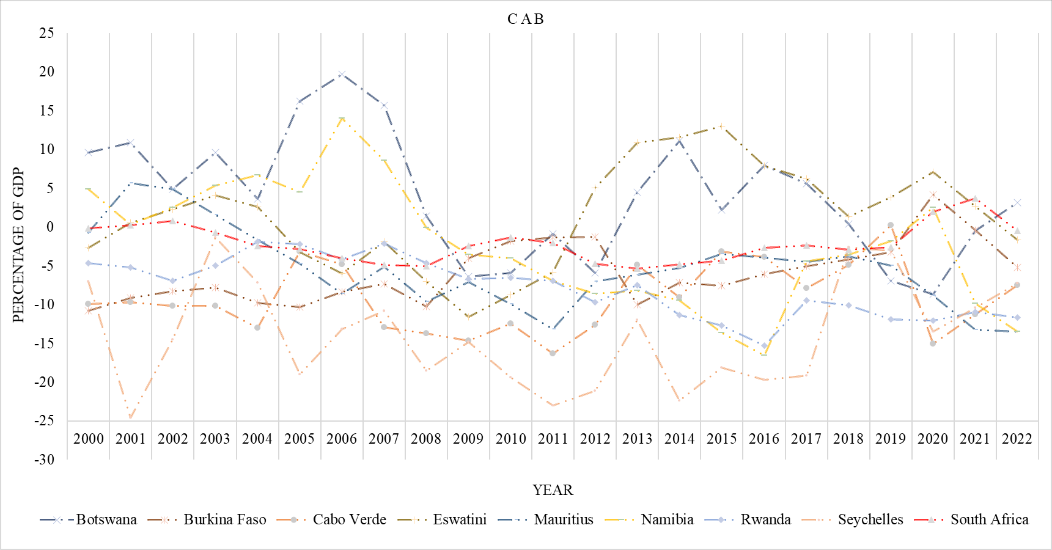


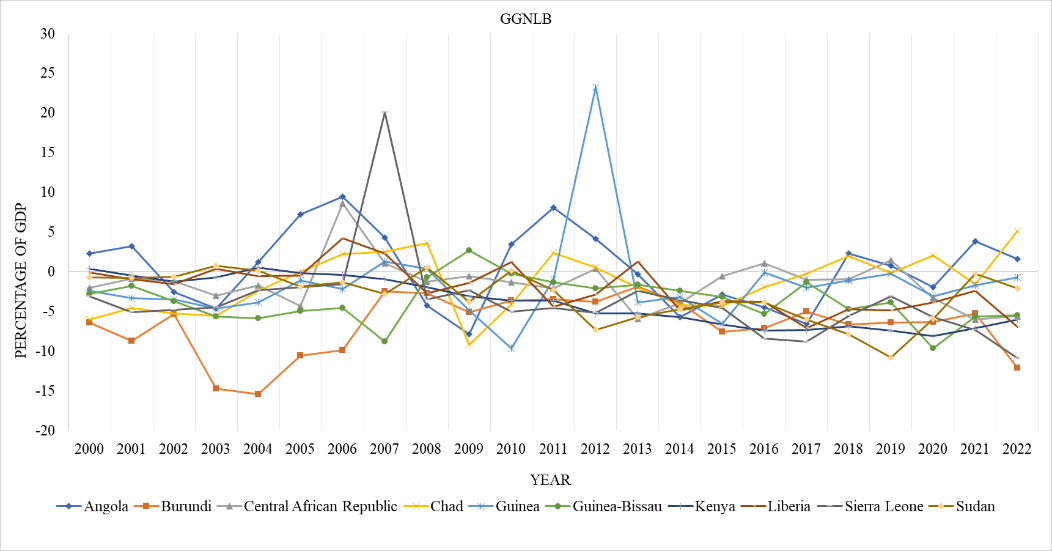

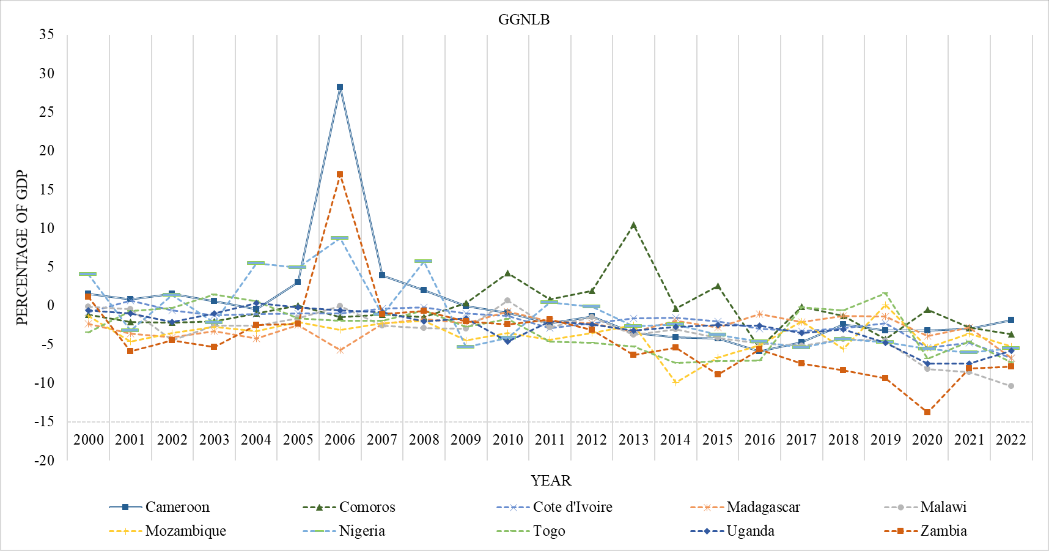

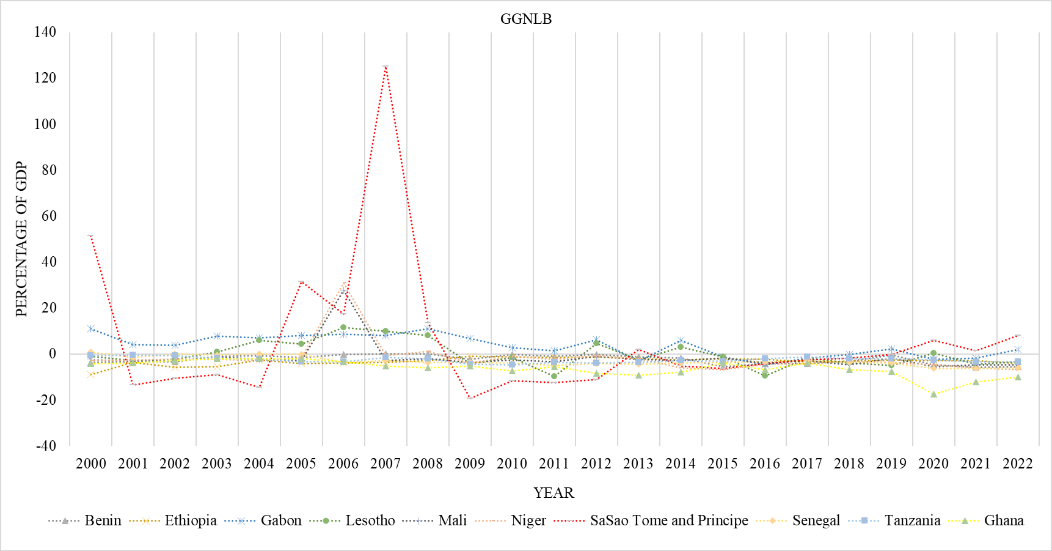

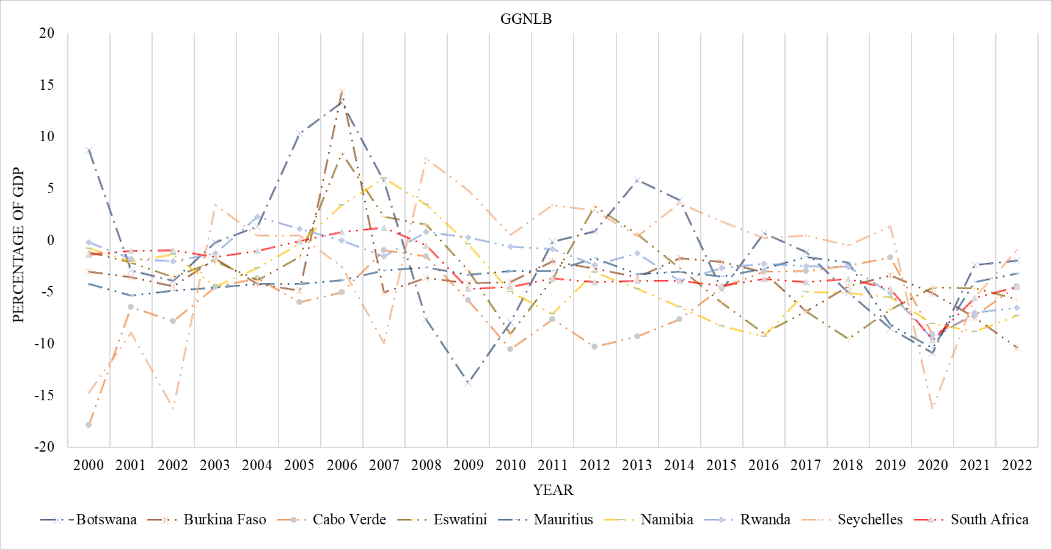


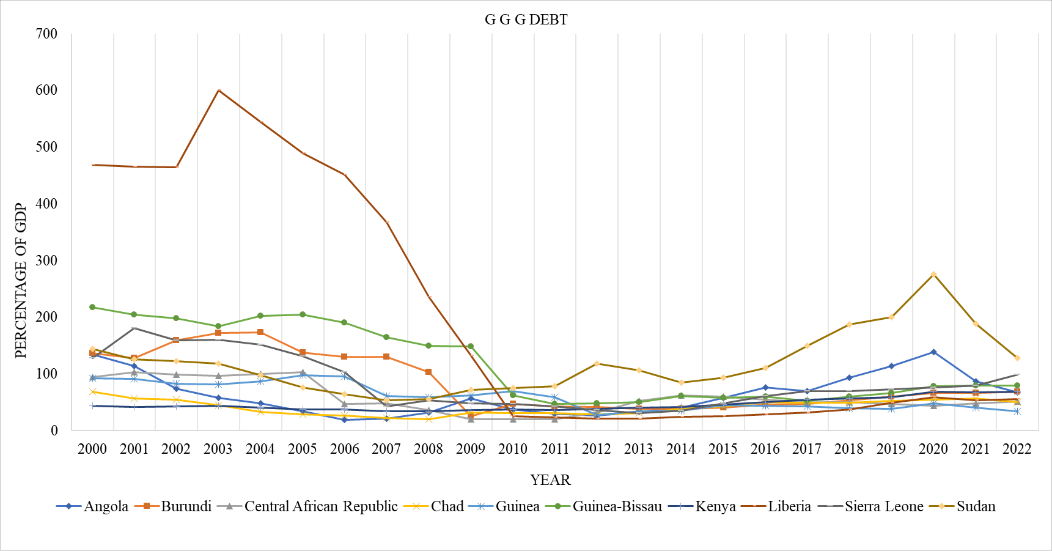

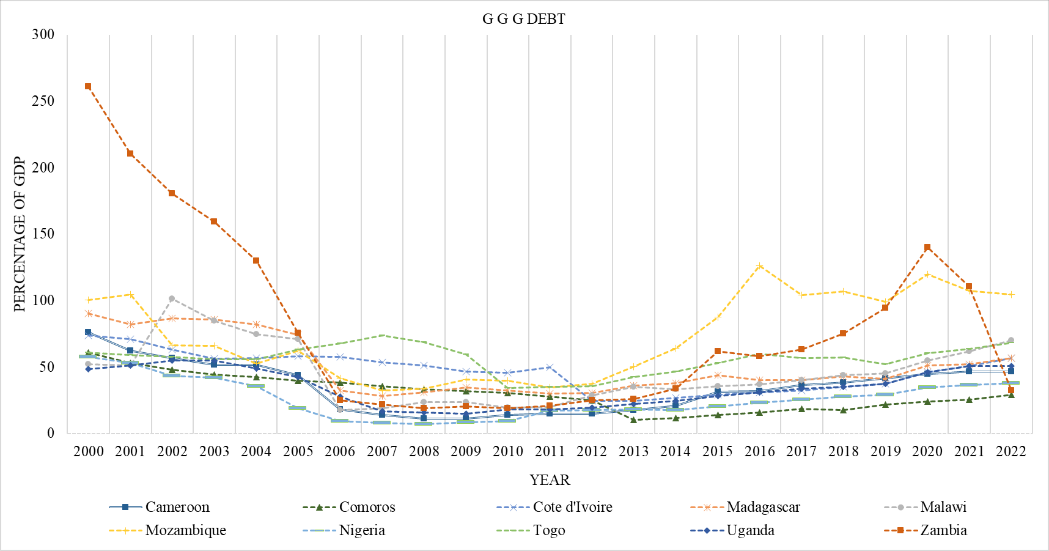

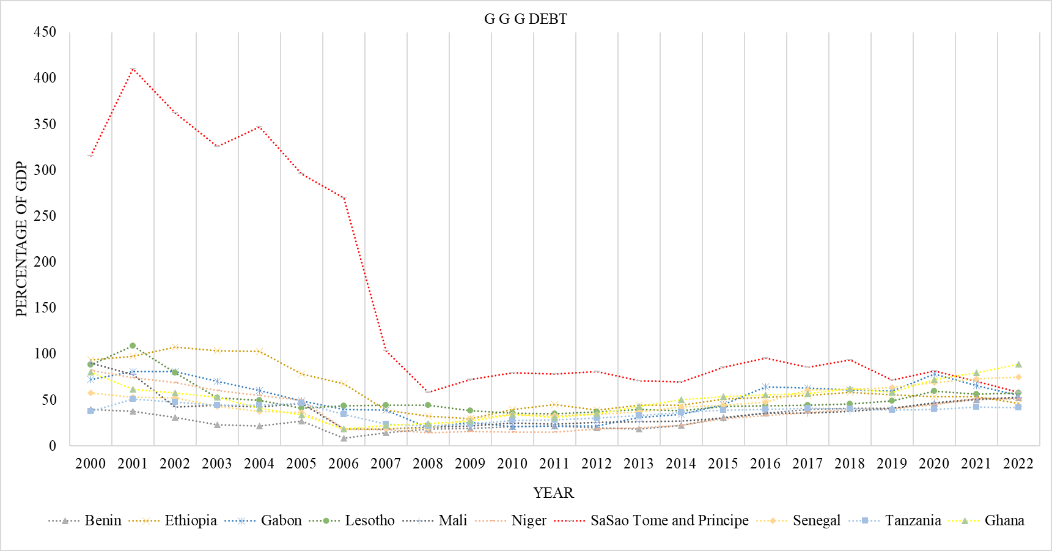

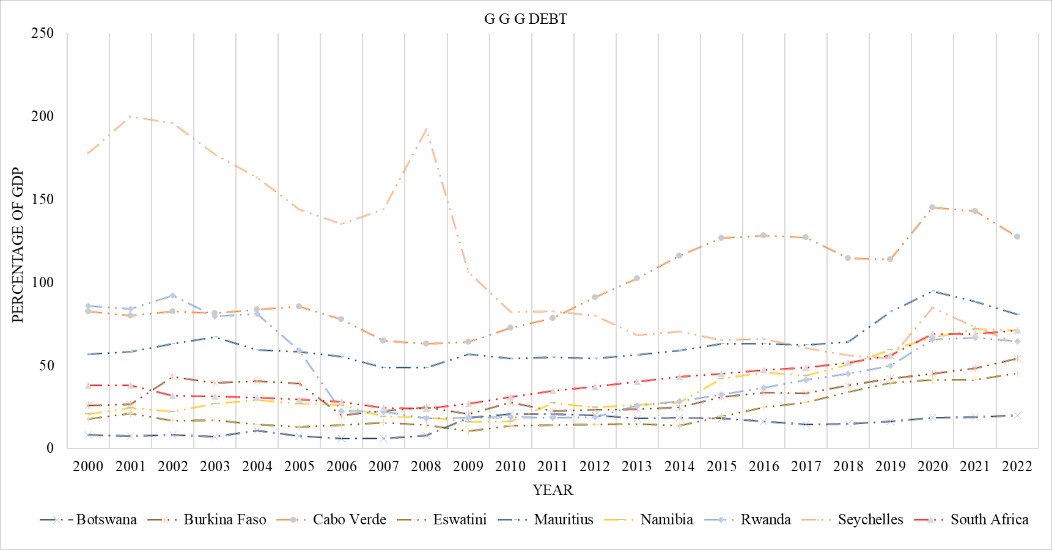


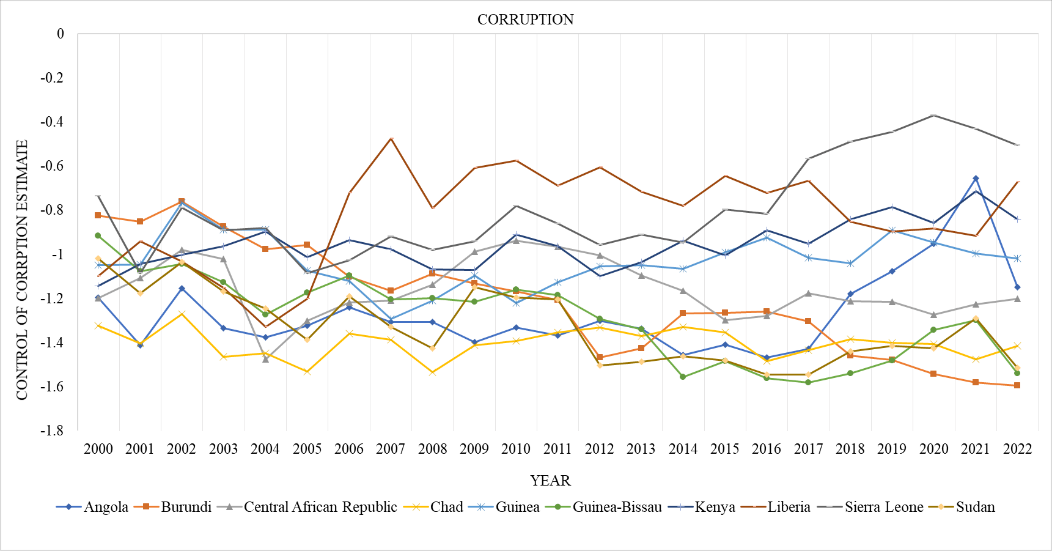

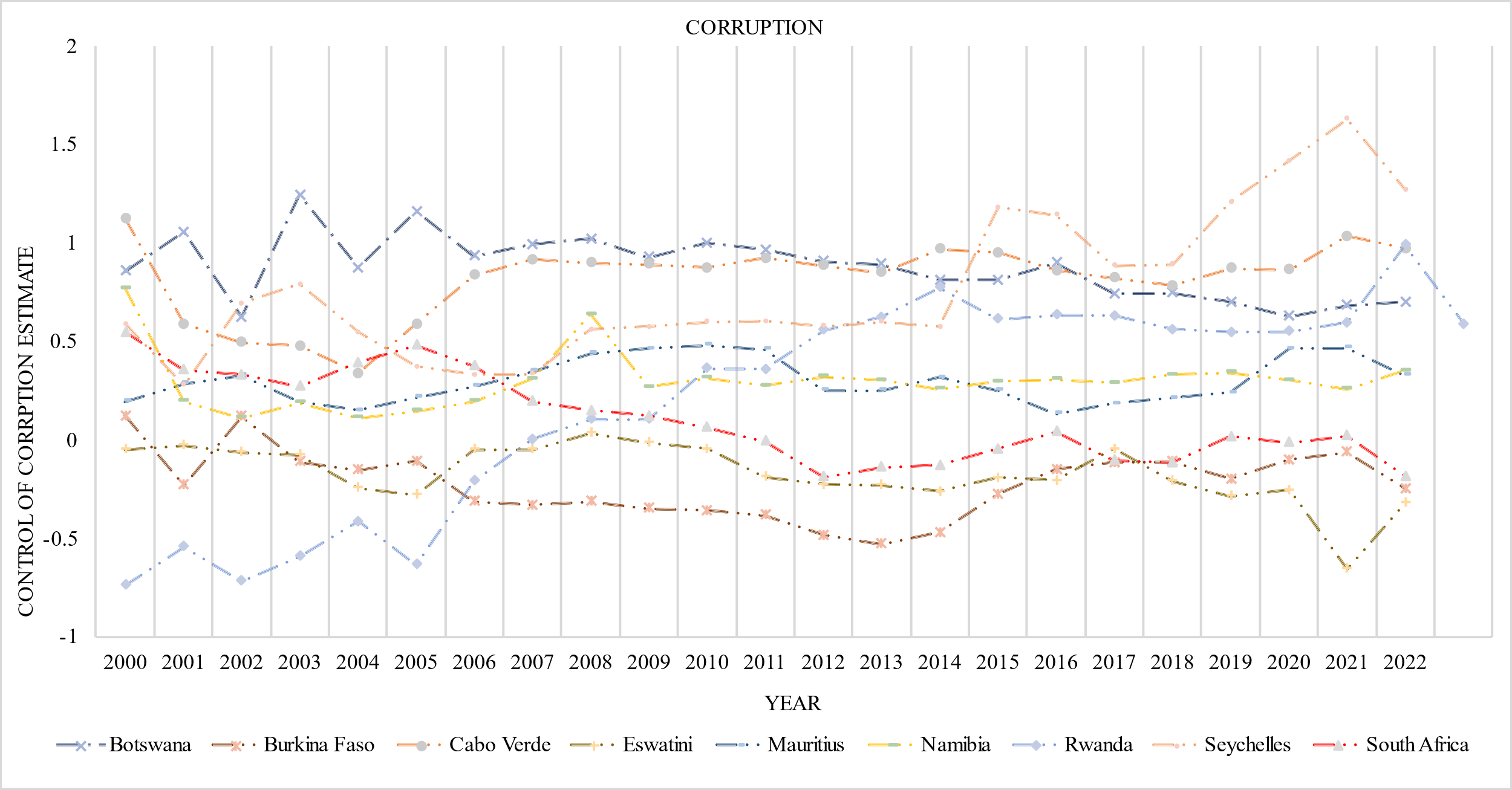


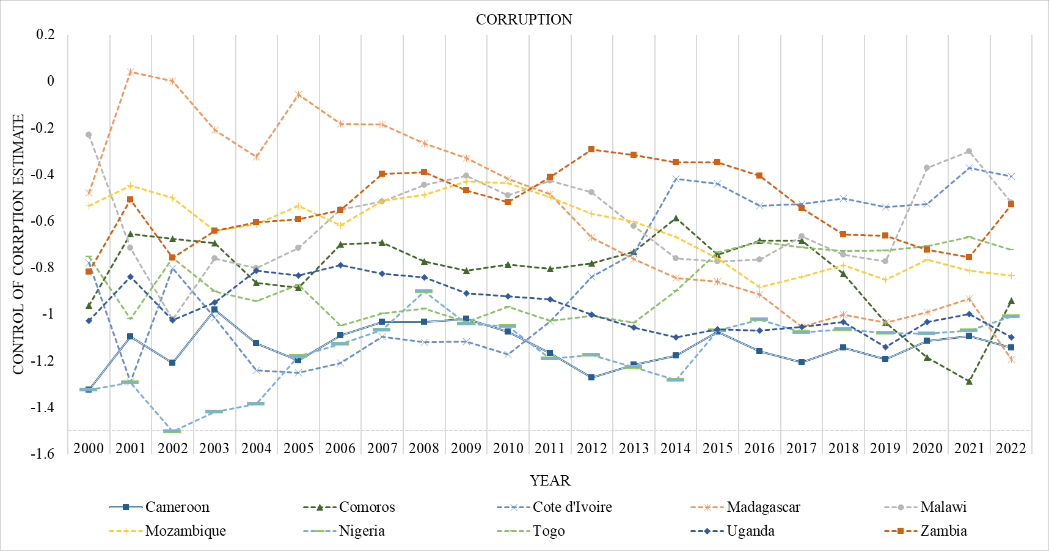

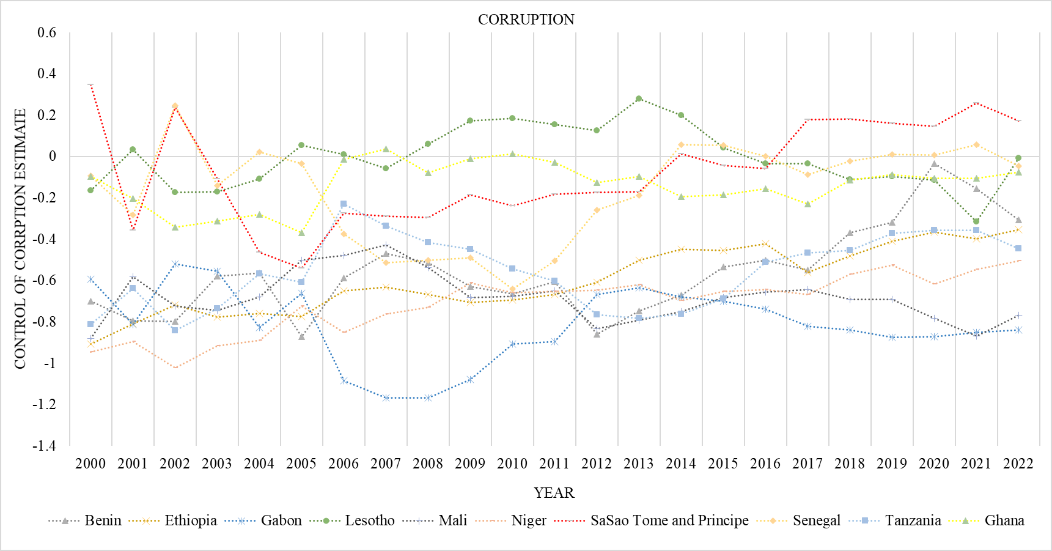


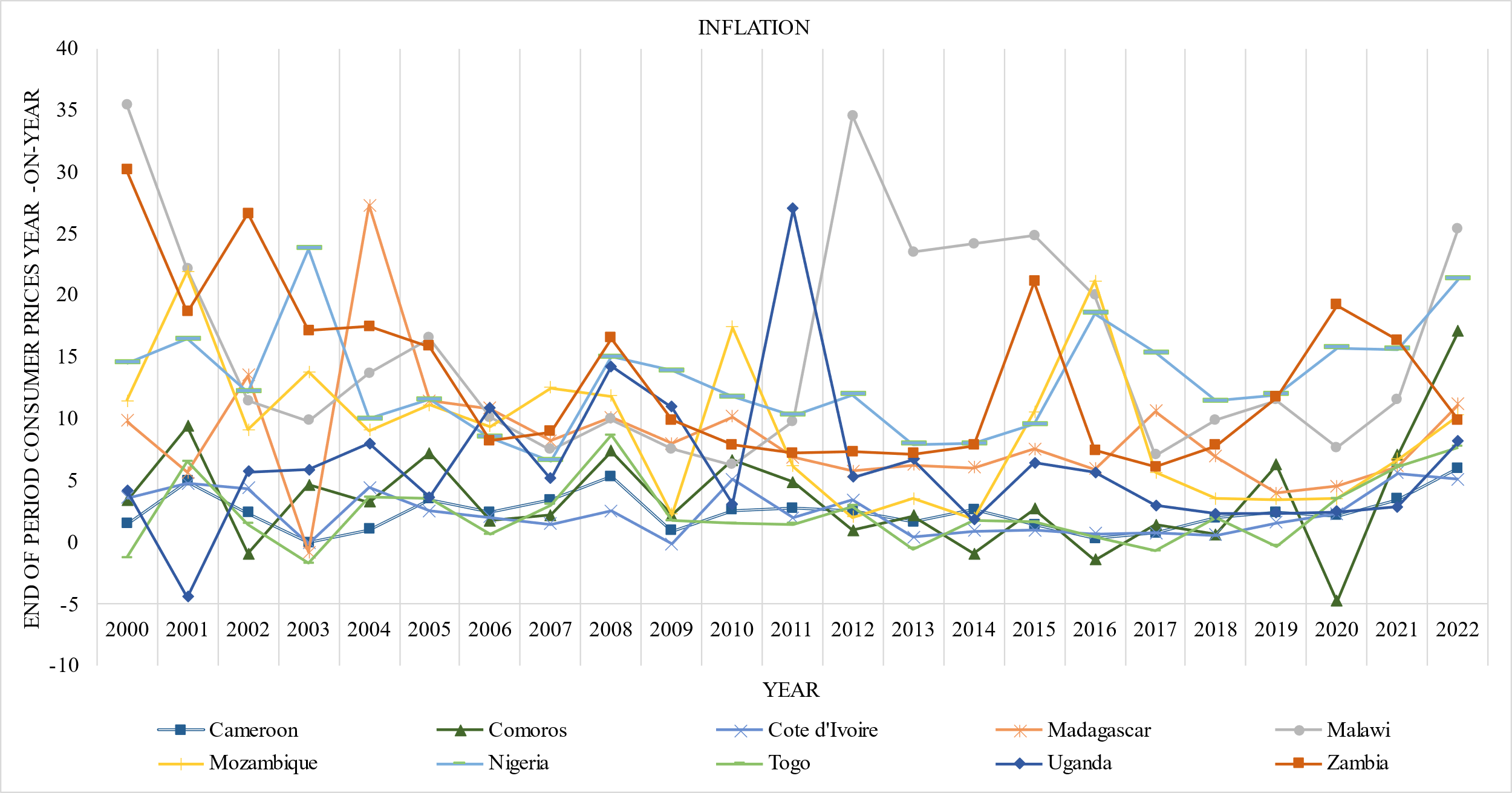

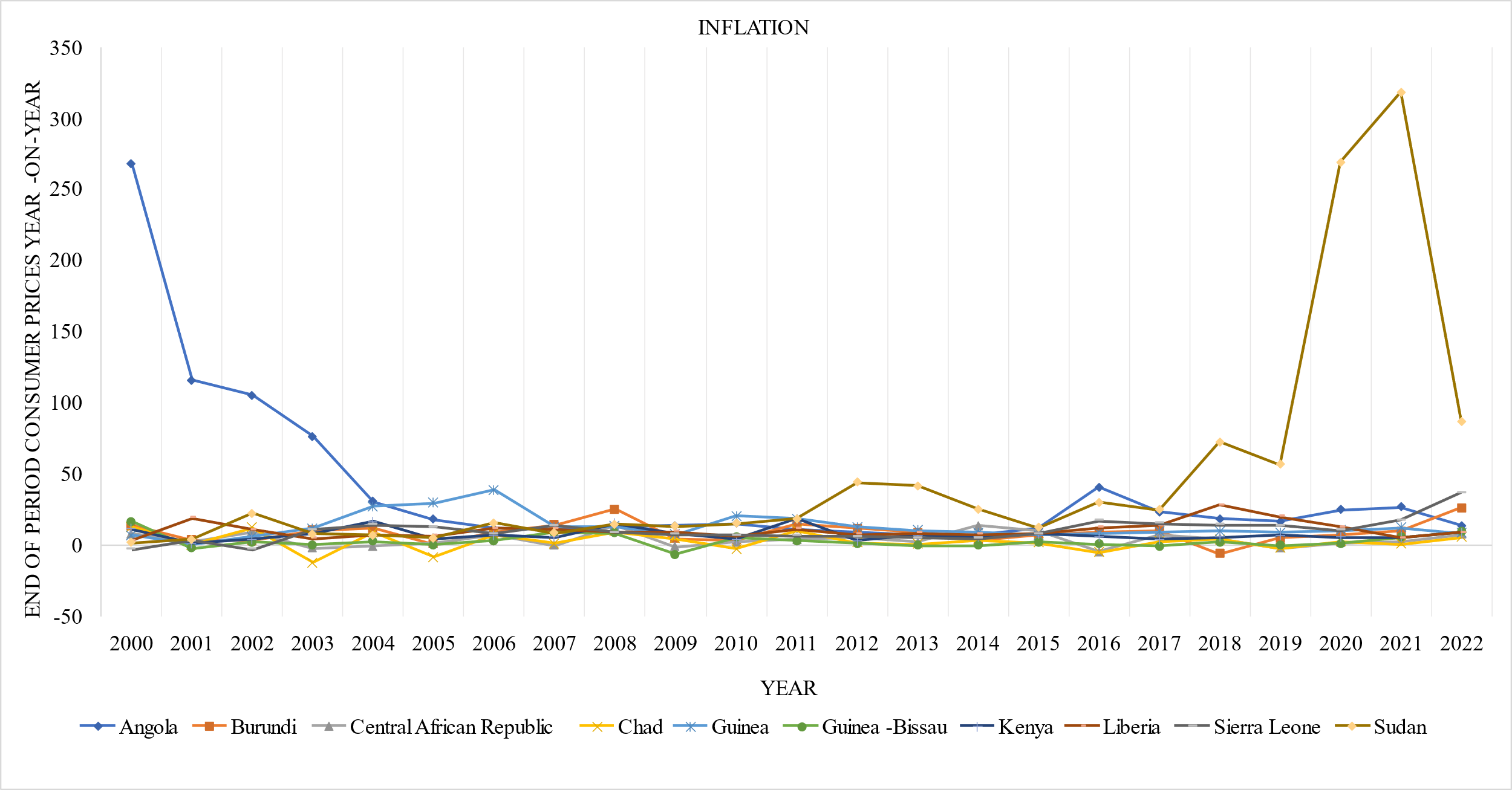


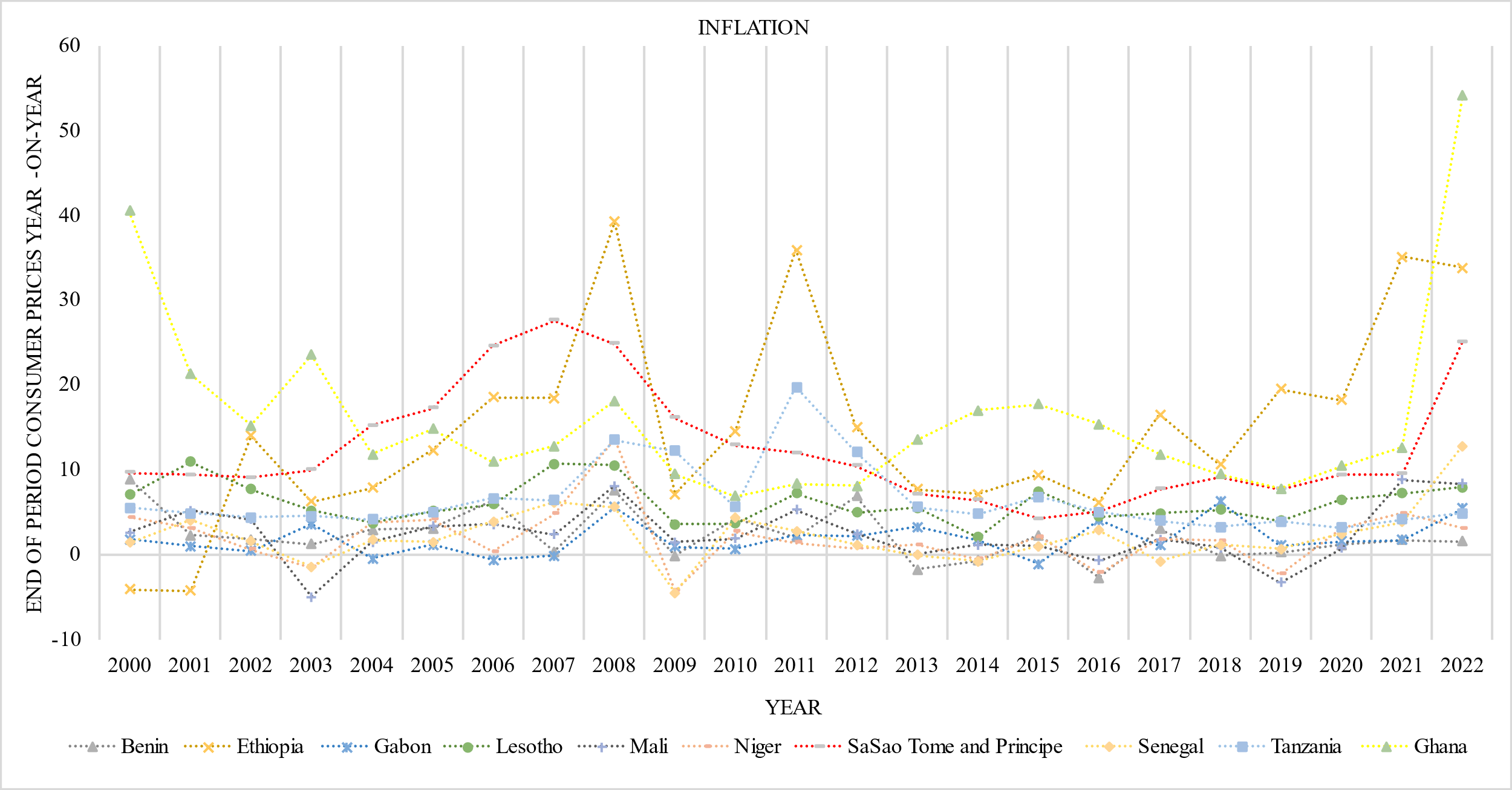


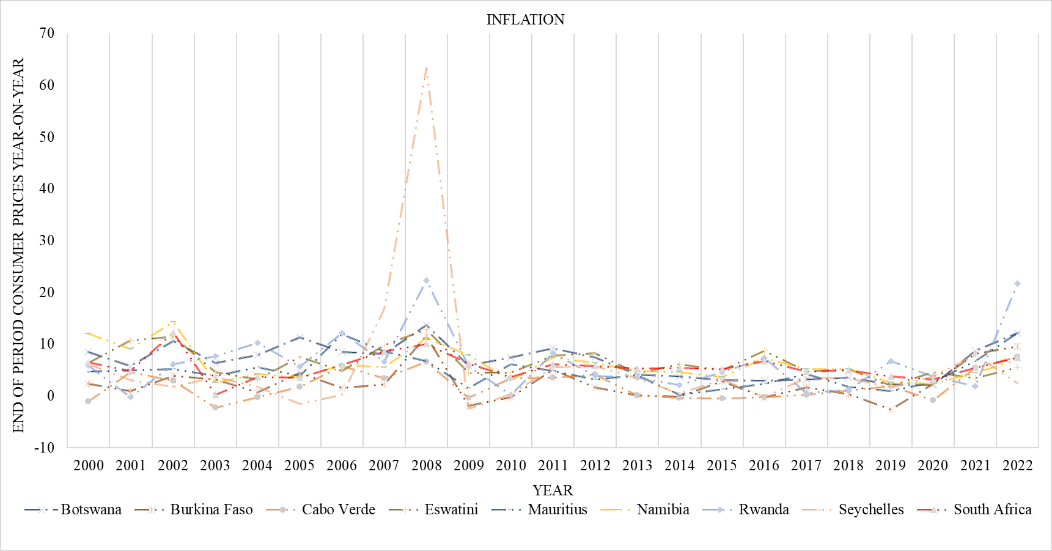


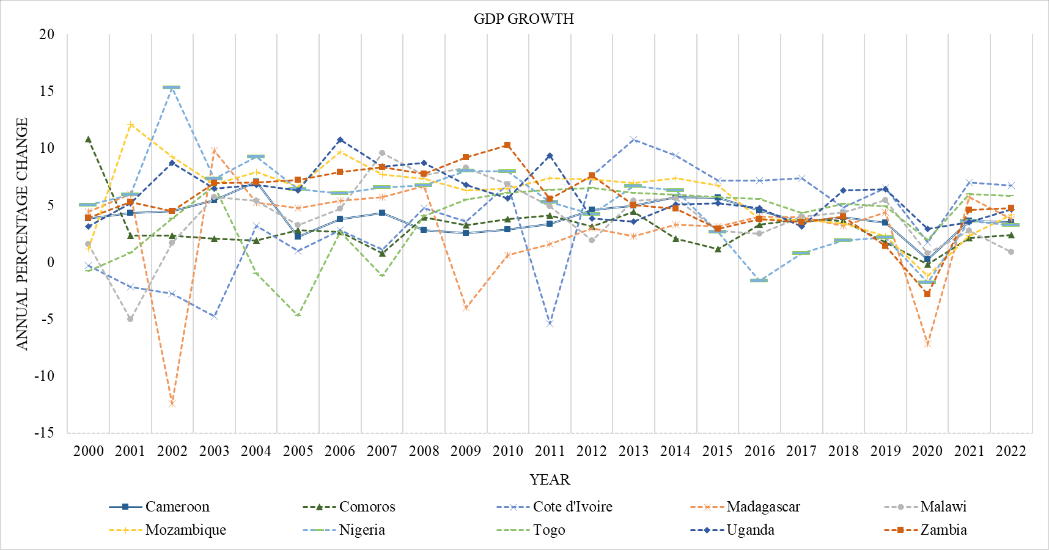

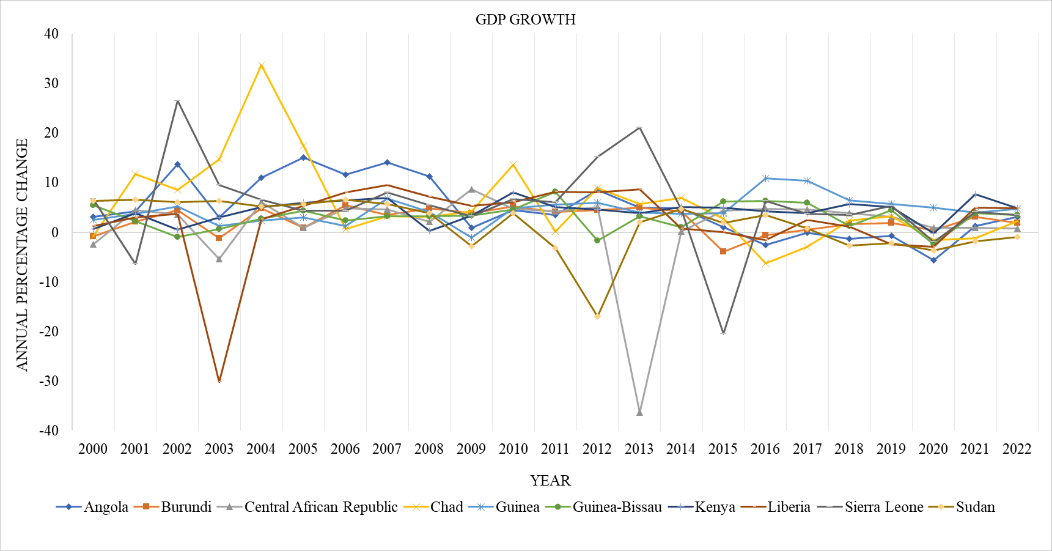


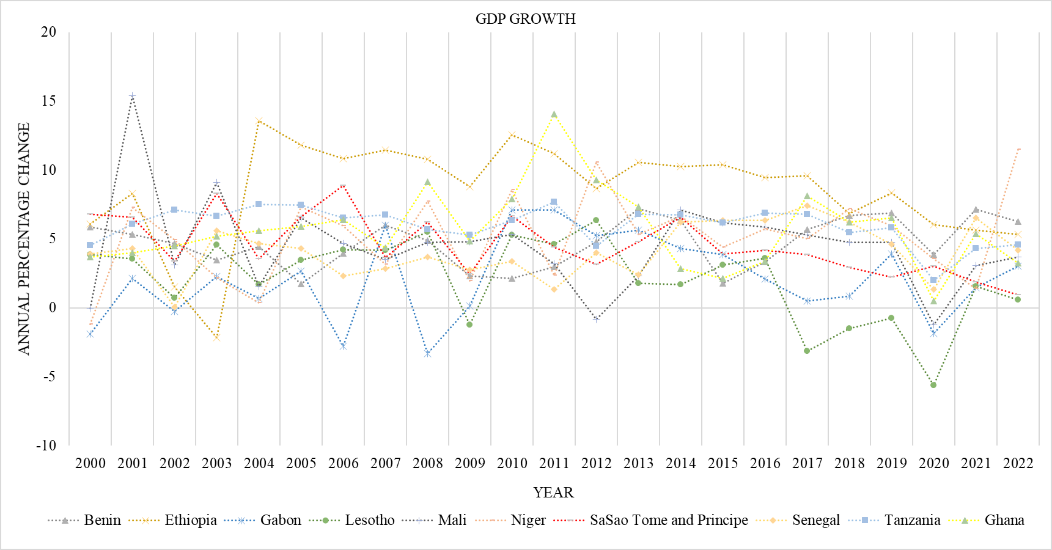

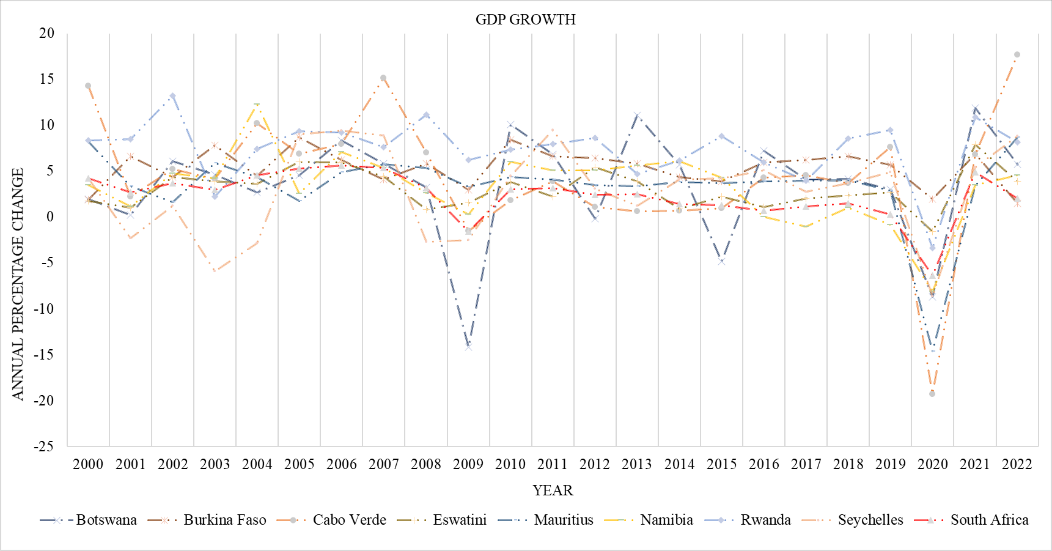

Supplement: S4 Appendix — (DOCX) [file pone.0307071.s004.docx]
