# Supplementary material for: Factors influencing IMF assistance in the Sub-Saharan African region
Source: PLoS One. 2024 Jul 16;19(7):e0307071. doi: 10.1371/journal.pone.0307071 (PMC11251602; doi:10.1371/journal.pone.0307071)
Supplement: S5 Appendix — (DOCX) [file pone.0307071.s005.docx]

**S5 Appendix: SSA countries predicted probability ranking of seeking IMF assistance.**

| **R** | **Country** | **IL** | **2000-2022** | **Country** | **IL** | **2017-2022** | **Country** | **IL** | **2019-2022** |
| --- | --- | --- | --- | --- | --- | --- | --- | --- | --- |
| **1** | Sudan | LI | 0.2613 | Sudan | LI | 0.4229 | Sudan | LI | 0.4443 |
| **2** | Liberia | LI | 0.2325 | Guinea-Bissau | LI | 0.3292 | Guinea-Bissau | LI | 0.3419 |
| **3** | Guinea-Bissau | LI | 0.2233 | Burundi | LI | 0.2863 | Burundi | LI | 0.3110 |
| **4** | Burundi | LI | 0.2011 | Liberia | LI | 0.2849 | Liberia | LI | 0.3015 |
| **5** | Sierra Leone | LI | 0.1997 | Sierra Leone | LI | 0.2370 | Malawi | LI | 0.2506 |
| **6** | Malawi | LI | 0.1966 | Malawi | LI | 0.2353 | Sierra Leone | LI | 0.2432 |
| **7** | CAR | LI | 0.1821 | Angola | LMI | 0.2243 | CAR | LI | 0.2286 |
| **8** | São Tomé | LMI | 0.1812 | Nigeria | LMI | 0.2178 | Nigeria | LMI | 0.2203 |
| **9** | Chad | LI | 0.1658 | CAR | LI | 0.2084 | Mozambique | LI | 0.2154 |
| **10** | Niger | LI | 0.1533 | Mozambique | LI | 0.2056 | Angola | LMI | 0.2138 |
| **11** | Angola | LMI | 0.1497 | Chad | LI | 0.1973 | Madagascar | LI | 0.2049 |
| **12** | Nigeria | LMI | 0.1420 | Madagascar | LI | 0.1785 | Cameroon | LMI | 0.1944 |
| **13** | Kenya | LMI | 0.1415 | Cameroon | LMI | 0.1756 | Zambia | LMI | 0.1929 |
| **14** | Togo | LI | 0.1377 | Niger | LI | 0.1727 | Chad | LI | 0.1881 |
| **15** | Mali | LI | 0.1350 | Lesotho | LMI | 0.1722 | Mauritius | UMI | 0.1876 |
| **16** | Zambia | LMI | 0.1346 | Zambia | LMI | 0.1693 | Comoros | LMI | 0.1822 |
| **17** | Senegal | LMI | 0.1323 | Gabon | UMI | 0.1673 | Lesotho | LMI | 0.1817 |
| **18** | Cote d'Ivoire | LMI | 0.1312 | Namibia | UMI | 0.1623 | Namibia | UMI | 0.1775 |
| **19** | Burkina Faso | LI | 0.1311 | South Africa | UMI | 0.1615 | Niger | LI | 0.1773 |
| **20** | Guinea | LMI | 0.1286 | Mauritius | UMI | 0.1568 | South Africa | UMI | 0.1750 |
| **21** | Mozambique | LI | 0.1280 | Kenya | LMI | 0.1558 | Senegal | LMI | 0.1722 |
| **22** | Cameroon | LMI | 0.1264 | Uganda | LI | 0.1512 | Gabon | UMI | 0.1688 |
| **23** | Benin | LMI | 0.1179 | Comoros | LMI | 0.1487 | Kenya | LMI | 0.1666 |
| **24** | Madagascar | LI | 0.1174 | Togo | LI | 0.1447 | Uganda | LI | 0.1570 |
| **25** | Gabon | UMI | 0.1113 | Senegal | LMI | 0.1438 | Togo | LI | 0.1500 |
| **26** | Lesotho | LMI | 0.1105 | Mali | LI | 0.1434 | São Tomé | LMI | 0.1441 |
| **27** | Uganda | LI | 0.1082 | São Tomé | LMI | 0.1425 | Mali | LI | 0.1410 |
| **28** | Comoros | LMI | 0.1038 | Burkina Faso | LI | 0.1304 | Ghana | LMI | 0.1409 |
| **29** | Namibia | UMI | 0.0949 | Guinea | LMI | 0.1286 | Cabo Verde | LMI | 0.1408 |
| **30** | Ethiopia | LI | 0.0906 | Cabo Verde | LMI | 0.1233 | Burkina Faso | LI | 0.1376 |
| **31** | South Africa | UMI | 0.0899 | Ghana | LMI | 0.1134 | Guinea | LMI | 0.1261 |
| **32** | Cabo Verde | LMI | 0.0885 | Cote d'Ivoire | LMI | 0.1064 | Ethiopia | LI | 0.1081 |
| **33** | Ghana | LMI | 0.0868 | Benin | LMI | 0.1060 | Cote d'Ivoire | LMI | 0.1081 |
| **34** | Tanzania | LMI | 0.0843 | Tanzania | LMI | 0.0979 | Tanzania | LMI | 0.1032 |
| **35** | Mauritius | UMI | 0.0808 | Ethiopia | LI | 0.0912 | Benin | LMI | 0.0875 |
| **36** | Seychelles | HI | 0.0587 | Eswatini | LMI | 0.0731 | Eswatini | LMI | 0.0761 |
| **37** | Eswatini | LMI | 0.0531 | Botswana | UMI | 0.0500 | Botswana | UMI | 0.0631 |
| **38** | Rwanda | LI | 0.0440 | Rwanda | LI | 0.0494 | Rwanda | LI | 0.0575 |
| **39** | Botswana | UMI | 0.0263 | Seychelles | HI | 0.0396 | Seychelles | HI | 0.0425 |

Note: R=Rank, IL-Income Level, HI- High Income, UMI – Upper Middle Income, LMI – Lower Middle Income, LI- Lower Income, CAR -Central African Republic.
